# Hydrogen peroxide modification affects the structure and physicochemical properties of dietary fibers from white turnip (*Brassica Rapa* L.)

**DOI:** 10.1038/s41598-020-80410-1

**Published:** 2021-01-13

**Authors:** Qi Gao, Xue-jie Zhou, Rui Ma, Han Lin, Jia-le Wu, Xue Peng, Masaru Tanokura, You-lin Xue

**Affiliations:** 1 College of Light Industry, Liaoning University, No. 66 Chongshan Middle Road, Huanggu District, Liaoning Province, Shenyang, 110036 People’s Republic of China; 2 Department of Applied Biological Chemistry, Graduate School of Agricultural and Life Sciences, The University of Tokyo, 1-1-1 Yayoi, Bunkyo-ku, Tokyo, 113-8657 Japan; 3 Party School of Liaoning Provincial Party Committee, Shenyang, 110161 People’s Republic of China

**Keywords:** Dietary carbohydrates, Chemical modification

## Abstract

Turnip (*Brassica rapa* L.) is widely consumed as a vegetable and traditional Chinese medicine with high dietary fiber content. Soluble dietary fiber (SDF) and insoluble dietary fiber (IDF) were obtained from white turnips, and the IDF was modified with alkaline hydrogen peroxide to obtain modified IDF (MIDF) and modified SDF (MSDF). The compositional, structural, and functional properties of the four samples were investigated. After modification, the modified dietary fibers (MDFs) showed smaller particle sizes and lower contents of pectin and polyphenol than those of unmodified dietary fibers (DFs) The results of scanning electron microscopy (SEM), Fourier transformed infrared (FT-IR) spectroscopy, X-ray diffraction (XRD) and differential scanning calorimetry (DSC) showed that compared to the DFs, the MDFs were smaller and had more exposed hydroxyl groups. Analysis of the microrheological behaviors showed that the MDFs had higher viscosity than that of the DFs, with a looser structure for the MSDF and a stable structure for the MIDF. Therefore, due to structural changes, the physical and functional properties of the MDFs were improved compared to those of the unmodified DFs. Pearson correlation analysis showed that the particle size was positively correlated with the pectin content. The water holding capacity (WHC), oil adsorption capacity (OAC) and water swelling capacity (WSC) showed positive correlations with each other. This work indicated that white turnip could be a potential new source of DFs, which presented desirable functional properties after modification.

## Introduction

The cultivation of turnip (*Brassica Rapa* L.) has a long history. As one kind of Cruciferae, turnip is adapted to the adverse environments of the Qinghai-Tibetan Plateau and possesses feeding, edible, and pharmaceutical value, which is highly appreciated as vegetable and traditional Chinese medicine^1,2^. In addition, it is consumed in enormous quantities not only in Tibet, China, but also throughout the world due to its rich phytochemicals, including glucosinolates, isothiocyanates, polysaccharides, triterpenoids, polyphenols and flavonoids^3,4^. Pharmacological investigation on turnip revealed the antitumor, antihypertensive, antidiabetic, antioxidant, antiinflammatory, hepatoprotective, and nephroprotective effects^4^. Glucosinolates and isothiocyanates are the main constituents for the protective effect against cancers, while, flavonoids and phenolics are corresponding to the antioxidant effects^4,5^.

According to the USDA (United States Department of Agriculture) National Nutrient Database (2015), fresh turnip contains 3.5% dietary fiber (DF)^6^. Traditionally, DF has been defined as the varieties of polysaccharide and lignin in the diet that are indigestible by endogenous secretions in the digestive tract^7^. DF is a mixture of cellulose, hemicellulose, gums, lignin and pectin, and can be divided into soluble dietary fiber (SDF) and insoluble dietary fiber (IDF)^8^. DF has certain physical characteristics and physiological and bioactive properties, such as capacity to form gels, fermentability, antioxidant activity, viscosity, and the ability to reduce the risk of colon cancer, hypertension, coronary heart disease, diabetes, asthma and obesity, which can be readily applied to food systems as a thickener, emulsifier, stabilizer, and fat replacer^9,10^.

Usually, DF can be modified by chemical, physical and biological approaches. Chemical modifications (e.g. acid hydrolysis, oxidation, etherification, esterification and cross-linking) may change the DF component as well as the physicochemical properties with extensive washing of material and generating polluent residues^11^. Physical methods (e.g. high-pressure homogenization, blasting extrusion, ultrafine grinding, and micro-fluidization) improve the physico-chemical and functional properties of DF by decreasing particle size rather than by increasing SDF content^12^. Biological methods are expensive because they require purified enzymes (e.g. cellulose, hemicellulase, xylanase, amylase, amyloglucosidase alcalase, pepsin, papain, and trypsin) or bacterial strains^13^.

This study focused on the extraction of DFs from white turnips and promoting their functional properties by chemical modification using alkaline hydrogen peroxide, which could react with hemicellulose and lignin to form water-soluble molecules with lower molecular masses than those of the original molecules^14^. The structural differences between DFs and modified DFs (MDFs) from white turnip were determined by particle size analysis, scanning electron microscopy (SEM), Fourier transform infrared (FT-IR) spectroscopy, X-ray diffraction (XRD), differential scanning calorimetry (DSC) and microrheological behavior analysis. Furthermore, the capacities of water holding, water swelling, oil adsorption, cation exchange and cholesterol absorption of the samples were also determined to explore the effects of the modification and the potential commercial value and health benefits.

## Results and discussion

### Basic components, color and granularity analysis

Alkaline hydrogen peroxide could modify the structure of hemicellulose to increase the water solubility, resulting in many differences between the MDFs and DFs^15^. The basic components of the DFs are shown in Table 1. The modification treatment resulted in a significant reduction in the pectin content, and the MIDF had the lowest pectin content. For polyphenol, the content decreased after modification, and that in MIDF was the lowest. The protein content of the DF samples was low, and the lowest values were detected in IDF and MIDF. Based on the color measurements, MDFs were obviously whiter than DFs, and MSDF was the whitest, followed by MIDF, IDF and SDF, which was caused by the bleaching effect of hydrogen peroxide^16^. The results of particle size analysis showed that modification reduced the particle size.

**Table 1:** The yield, basic content, color, WHC, OAC, WSC, crystallinity, particle size, and CAB of DFs from white turnip.

	SDF	MSDF	IDF	MIDF
Yield (%)	20.06 ± 1.77b	11.49 ± 2.50d	51.35 ± 1.89a	19.03 ± 1.99c
Pectin (g/100 g)	6.65 ± 0.50a	1.62 ± 0.35c	6.10 ± 0.65b	0.90 ± 0.42d
Polyphenol (g/100 g)	2.35 ± 0.33a	2.11 ± 0.12b	2.35 ± 0.30a	0.43 ± 0.21c
Protein (g/100 g)	1.70 ± 0.34a	1.72 ± 0.40a	1.05 ± 0.45b	0.97 ± 0.26c
L*	79.22d	98.92a	81.6c	90.27b
a*	8.42a	− 0.02d	5.16b	3.47c
b*	20.85a	7.85d	19.02c	19.3b
$\Delta {\mathrm{E}}$	–	1.74b	–	5.29a
WHC (g/g)	–	–	12.75 ± 0.01a	13.65 ± 0.01a
OAC (g/g)	3.29 ± 0.01b	3.71 ± 0.01a	2.29 ± 0.01d	2.41 ± 0.02c
WSC (mL/g)	–	–	5.67 ± 0.06a	5.97 ± 0.05a
Crystallinity (%)	34.19 ± 0.37a	14.84 ± 0.42c	6.76 ± 0.35d	18.71 ± 0.56b
Particle size (d, nm)	488.8 ± 25.73c	397.1 ± 47.99d	3961 ± 101.8a	2810 ± 68.59b
CAB (mg/g)	9.91 ± 0.36b	10.15 ± 0.75a	9.78 ± 0.53c	9.82 ± 0.61bc

Values are the means ± SD of triplicate determinations. The values with a–e indicate significant differences in the same rows, *P* < 0.05. “−” represents not detected.

### Structure analyses

#### SEM

**Figure 1:**
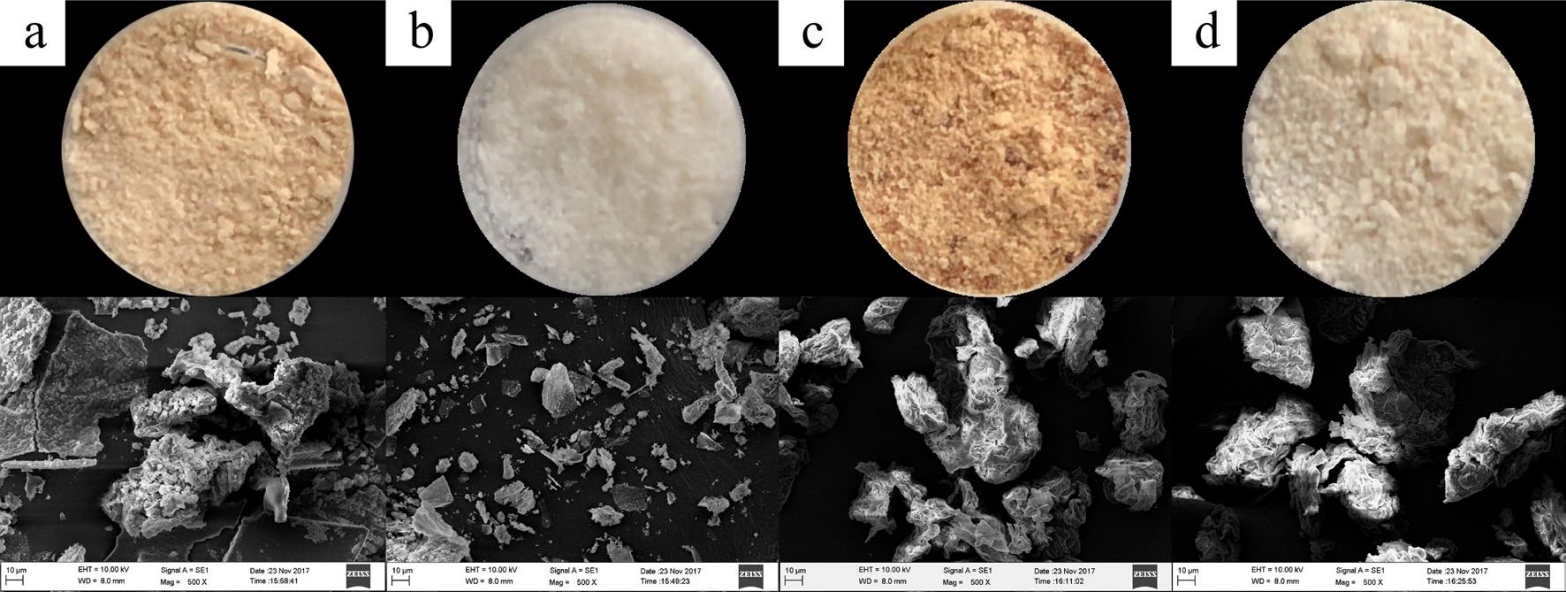
The powder and SEM images (500 ×) of (**a**) SDF, (**b**) MSDF, (**c**) IDF and (**d**) MIDF obtained from white turnip.

As shown in Fig. 1. characteristic honeycomb structure was observed in IDF, and more cracks and holes were detected in MIDF. The SDF particles were large with a rough surface, and a sheet structure was observed. The MSDF particles were relatively smaller with a smoother surface. The microstructure of MDFs was structurally relaxed, and surface wrinkles and cavities were observed. These changes indicated that the modification changed the structure, with some valleys and cracks formed, and the MSDFs were smoother than the SDFs, which resulted in an increase in the number of fiber particles^17^. At the same time, many heteropolysaccharides attached to the surface of the particles were detached, which increased the contact area between the hydroxyl groups and the environment^18^.

#### FT-IR

**Figure 2:**
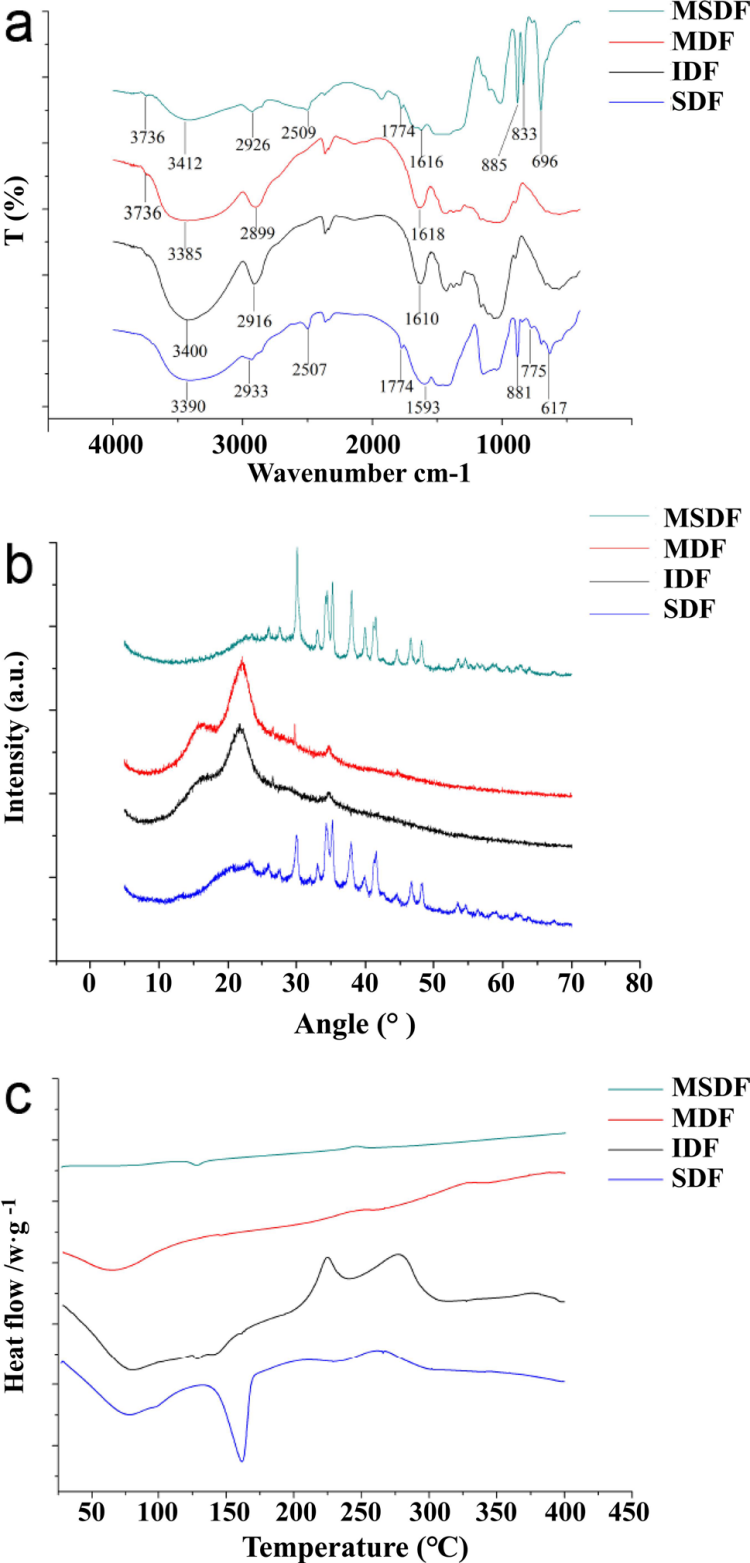
FTIR spectra (**a**), XRD measurements (**b**) and DSC analysis (**c**) of DFs from white turnip.

The functional groups and bonding information of the samples could be explained by the FT-IR spectra^17^. The FT-IR results are shown in Fig. 2a; the broad peak appearing at 3400 to 3200 cm^−1^ is an O–H stretching vibration derived from hydrogen-bonded alcohol or phenol^18^. The MSDF and MIDF showed characteristic peaks of free hydroxyl stretching vibrations at 3700–3500 cm^−1^, indicating that the content of free hydroxyl groups was increased after alkaline hydrogen peroxide modification^18^.

A typical –CH_2_ stretching vibration was observed at approximately 2920 cm^−1^, and a δC–H vibration of –CH_2_ was observed at 1465 ± 20 cm^−1^. In the fingerprint region, the SDF had a relatively weak peak at 775 cm^−1^, but this peak shifted to 833 cm^−1^ in MSDF, which indicated that the long chain of –CH_2_ groups was broken after the modification and that the content of oligosaccharides was increased^19^. SDF and IDF presented a characteristic absorption peak of the benzene ring at 1590–1620 cm^−1^, and the corresponding peaks were weakened in the MSDF and MIDF samples, which indicated that the original DFs contain more lignin, pectin and hemicellulose^20^.

A C=O stretching vibration was observed in SDF at 1774 cm^−1^. The corresponding peak of the MSDF was weakened, probably because the C=O groups, which are originally present in the fiber, were oxidized^21^.

#### XRD

Crystalline cellulose exhibits a sharp XRD peak, showing an extended crystal line and complete crystal surface^22^. As shown in Fig. 2b, broad peaks at 12.5–28° were observed for IDF and MIDF, and the peak position and width were not significantly different between IDF and MIDF, which indicated the ordered structure of the crystalline region on the cellulose^23^. As shown in Table 1, the crystallinity of MSDF (14.84%) was lower than that of SDF (34.19%), and the crystallinity of MIDF (18.71%) was higher than that of IDF (6.76%). According to a previous report, DFs consist of crystalline regions and amorphous regions, and the amorphous regions are composed of noncrystalline cellulose, hemicellulose, and lignin^24^. The amorphous regions of cellulose is easily destroyed, thus leading to increased relative ratio of crystalline cellulose after hydrolysis^23^. As a result, combined with the SEM results, the FT-IR results suggested that the modification damaged the amorphous regions of IDF (Fig. 1), thus leading to the conversion of the amorphous regions into water-soluble components^25^.

#### DSC

The exothermic peaks exhibited in the DSC curve illustrate the vaporization of volatile products and the thermal and oxidative decomposition of polymers^26^. Figure 2c shows the thermal properties of the DFs. In the first temperature range, 50–100 °C, an endothermic peak transition was observed with a maximal peak at approximately 75 °C, which may correspond to the evaporation of unbound water and the phase transition of pectin with sorbed water from crystalline to amorphous structure^27^. The 2nd endothermic transition of the DFs occurred at approximately 150 °C, which could be explained by the evaporation of bound water in the original DFs^28^. However, the intensity of the heat flow of DFs was significantly higher than that of MDFs, illustrating that DFs possessed a higher content of bound water. The exothermal peak at 230 °C might correspond to the pyrolysis peak of pectin^29^. The heat flow obtained from MDFs was lower than those from DFs, which was consistent with the pectin content (Table 1). The exothermal peaks at approximately 300 °C were probably the pyrolysis peaks of cellulose, hemicellulose or lignin^30^. For the MSDF, there is almost no peak after 300 °C, which corresponds to the decreased crystallinity of the MSDF (XRD results). In addition, a higher heat flux strength indicates lower thermal stability, which suggests that MDFs have higher thermal stabilities than DFs^31^.

### Physical properties

#### Water holding capacity (WHC), water swelling capacity (WSC) and oil adsorption capacity (OAC)

As shown in Table 1, the WHC and WSC of the MIDF increased slightly through modification, which exposed hydroxyl groups in the IDF structure^13^. Second, the hydrolysis of some heteropolysaccharides led to the exposure of more hydroxyl groups, allowing the accommodation of more water molecules. After the modification, the OAC increased, and the OAC of the SDFs was significantly higher than that of the IDFs^32^. The improvement of these properties indicated a good application prospect of MDFs in food processing.

### Cation exchange capability

**Figure 3:**
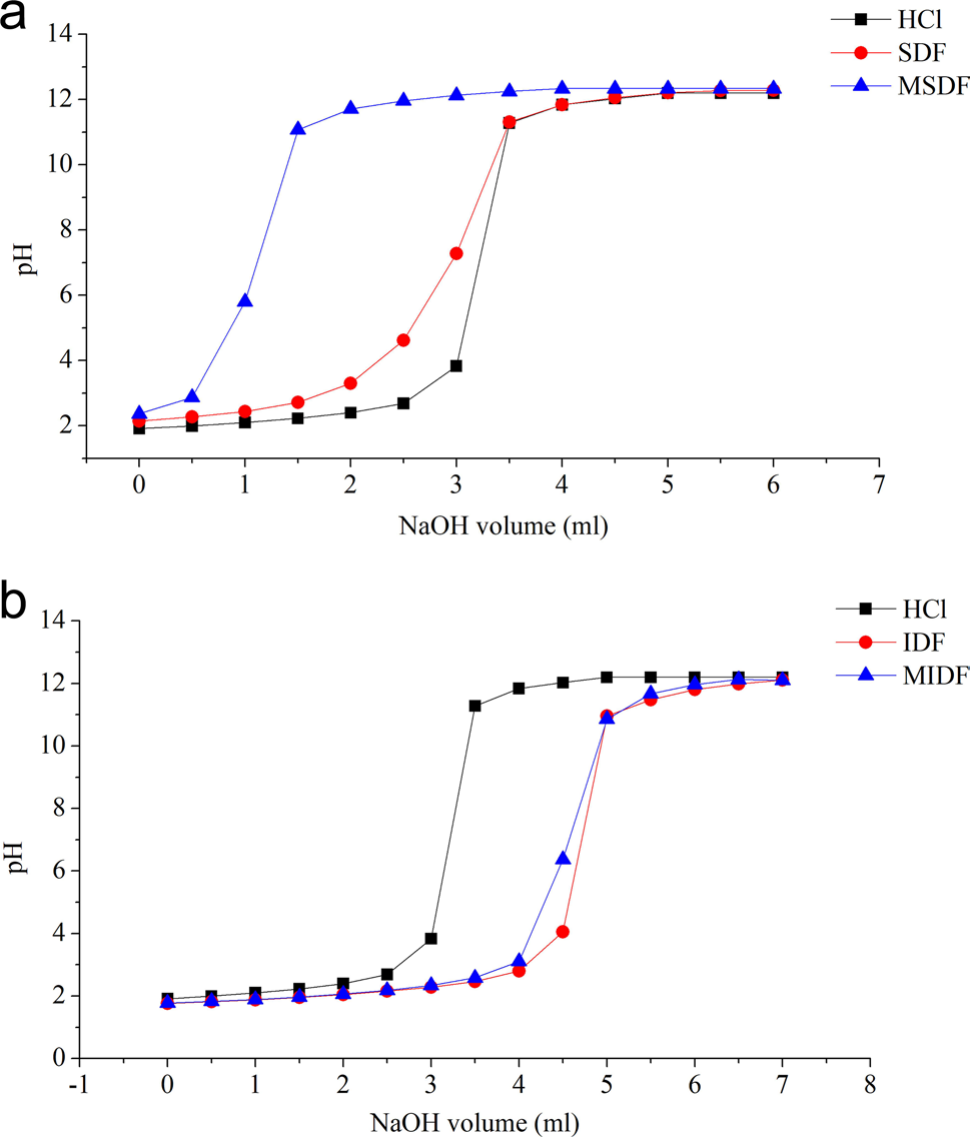
Cation exchange capability of (**a**) SDFs and (**b**) IDFs from white turnip.

The curves in Fig. 3a show that practically complete exchange of hydrogen ions occurs in 6 mL of 0.1 M NaOH. For the MSDF, the inflexion point was at 0.5–1.5 mL of NaOH. For the SDF, the inflexion point was in 2–3.5 mL of NaOH. This change indicates that the MSDF has a higher cation exchange capacity than that of the SDF. The curves in Fig. 3b show that a practically complete reaction of hydrogen ions occurs in 7 mL of 0.1 M NaOH. This change indicates that the IDF and MIDF showed delayed inflexion points. In conclusion, SDF and MSDF have strong cation exchange capacity, which can effectively control the drastic change in pH^33^.

### Microrheological properties

**Figure 4:**
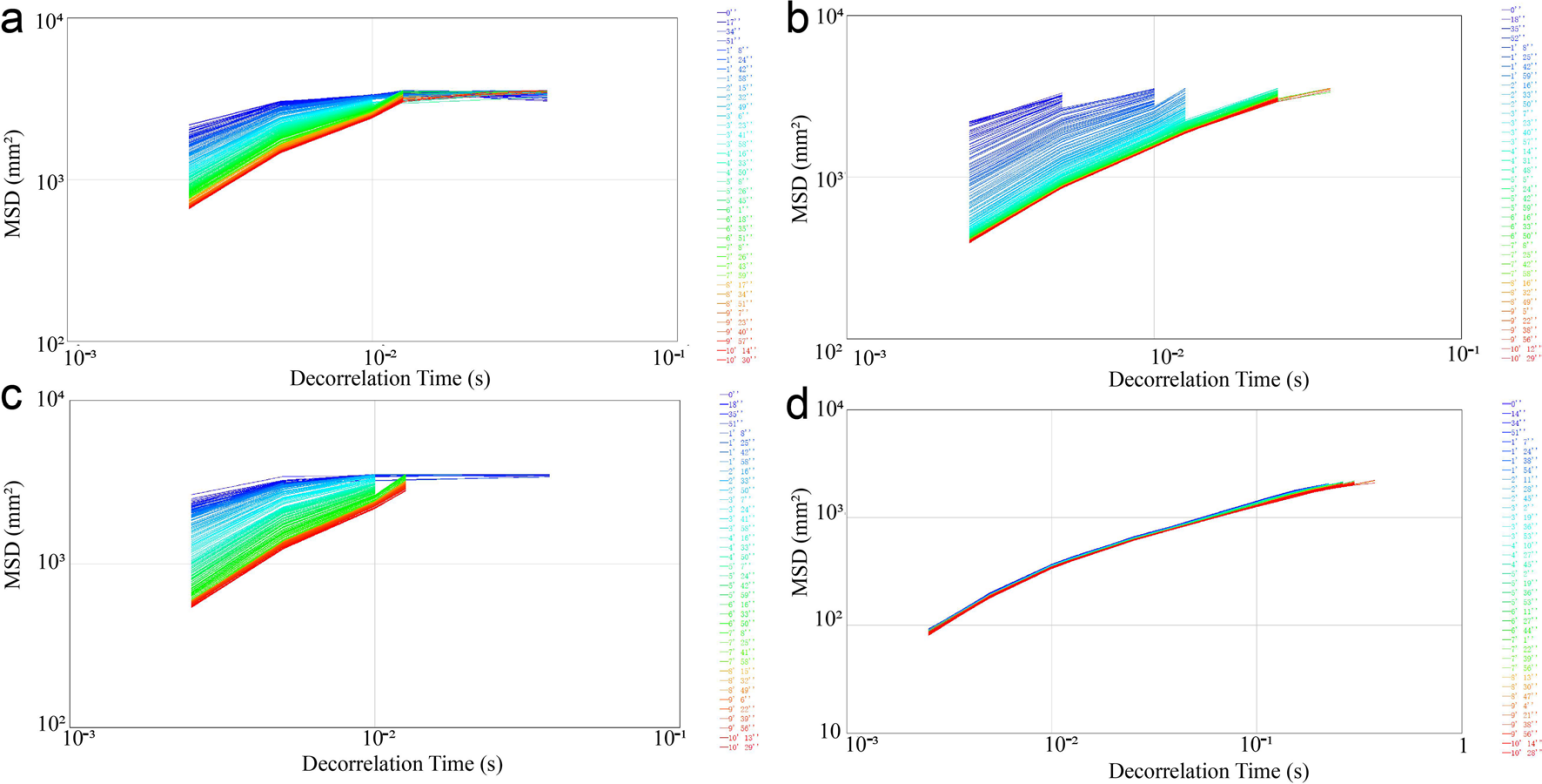
MSD of SDF (**a**), MSDF (**b**), IDF (**c**) and MIDF (**d**) from white turnip.

Compared with measuring shear rheology, the Rheolaser Lab technique does not modify the DF samples. The Brownian motion of droplets was monitored and interpreted in terms of microrheology^34^. The motion of the samples was measured, and the interactions of the particles were traced by microrheology, which were expressed as mean square displacements (MSDs) (Fig. 4). The MSD curves of the SDF (Fig. 4a) and IDF (Fig. 4c) were similar. The MSD curve of the MSDF (Fig. 4b) was not linear, indicating that MSDF had viscoelastic properties and that the droplets were free to move due to weak network interactions of droplets^35^. As shown in Fig. 4d, the MSD curves of the MIDF was linear, indicating that the network interactions of droplets was apparently structurally stable^35^. Therefore, Fig. 4a,b shows that the configuration of the MSDF apparently was structurally relaxed, and Fig. 4c,d shows that the configuration of the MIDF was structurally stablized. Alkaline hydrogen peroxide reacted with the IDF, which caused the loss of soluble fractions. Combined with the SEM and FT-IR results, the MSDF had smaller particle sizes and more free hydroxyl groups with relatively unstable structures compared to those of the other samples.

**Figure 5:**
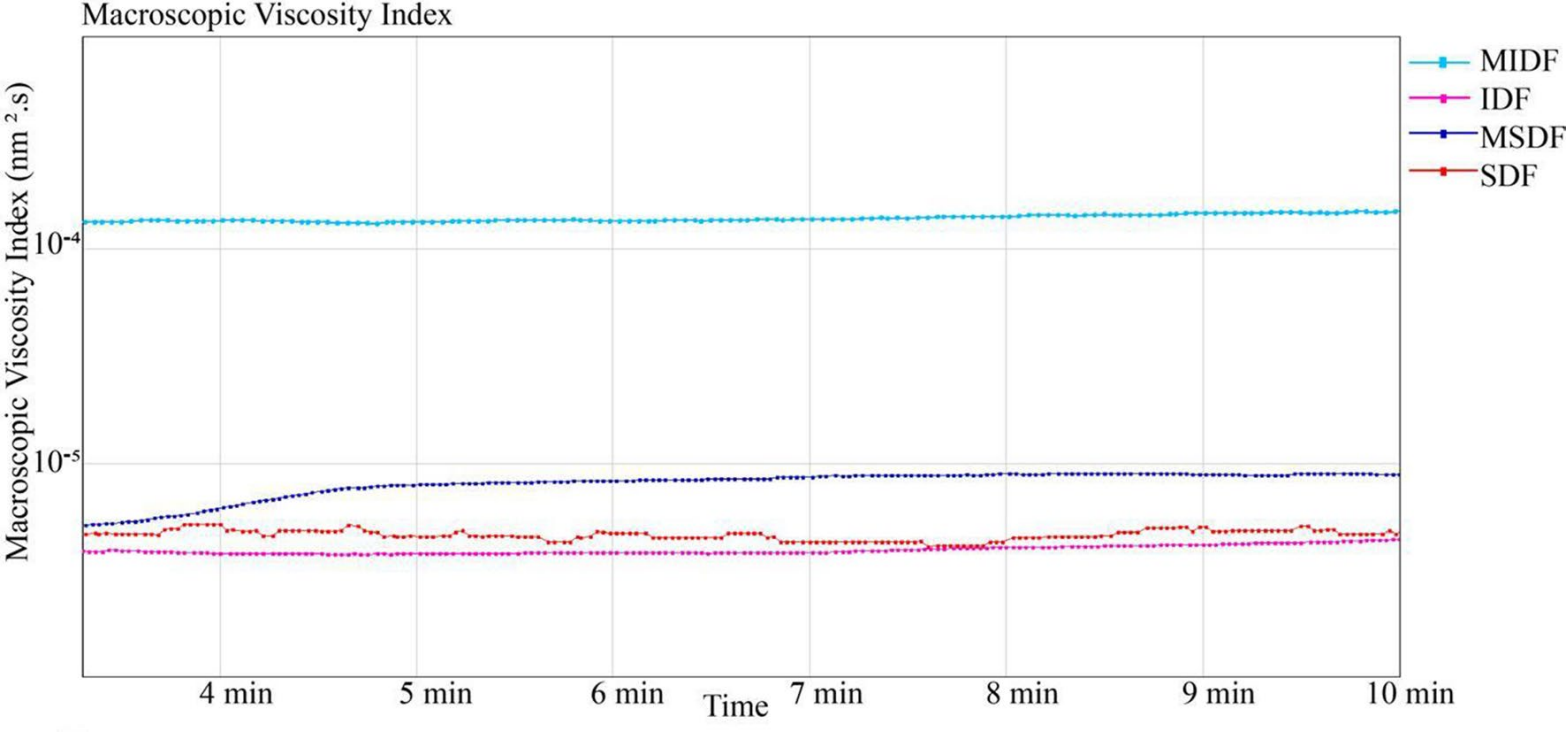
Macroscopic viscosity index of DFs from white turnip. The figure was generated by RheoSoft Master 1.4.0.0.

As shown in Fig. 5, the macroscopic viscosities of the DFs were represented by the macroscopic viscosity index (MVI) values, which corresponds to the inverse of the speed of the particles for long times^35^. The viscosity of the MIDF was the highest, followed by that of the MSDF, SDF, and IDF.

**Figure 6:**
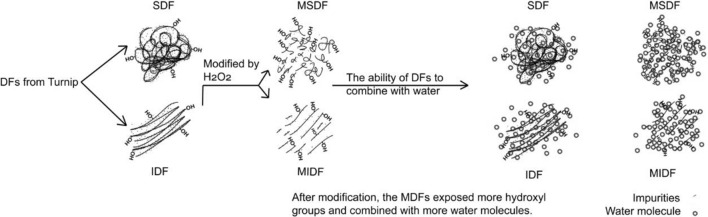
Possible mechanism for the better ability of MDFs to combine with water. The figure was generated by RheoSoft Master 1.4.0.0.

Due to the modification, the viscosity of MDFs increased. The peroxide-initiated free radicals reacted with the hydroxyl groups of the fiber and the matrix, and good fiber matrix adhesion along the interface occurred^21^. First, intermolecular hydrogen bond cleavage led to more exposure of polar groups in the molecule, and the charge on the surface of the molecule increased, thereby enhancing the electrostatic interaction among the molecules, as well as between the molecule and the solvent, and increasing the flow resistance of the solution. The increase in molecular surface charge also thickened the solvation layer of the molecule, which led to further dilation of the molecule and an increase in the viscosity of the solution^29^. Second, the modification treatment reduced the size of the fiber particles and smoothed them, which increased the effective area of the fiber surface and resulted in more particle interactions and an enhanced network structure with water molecules, resulting in a significant increase in apparent viscosity^36^. The difference between the MIDF and MSDF was that the crystallinity of the MIDF was higher and that its surface contained more valleys, fiber bundles and gullies (Fig. 1), which increased the effective area of the fiber surface and led to a higher viscosity of the MIDF than of the MSDF^37^. For the SDF and IDF, the former had a smaller particle size and higher crystallinity, which means a higher effective area of the fiber surface and greater number of fiber bundles, resulting in the viscosity of the SDF being higher than that of the IDF (Fig. 6).

### Cholesterol absorption ability (CAB)

As shown in Table 1, the MSDF (10.15 ± 0.75) had the strongest CAB, the SDF (9.91 ± 0.36) performed better than the MIDF (9.82 ± 0.61), and the IDF (9.78 ± 0.53) showed the lowest CAB. From the SEM images (Fig. 1), many valleys were cracked or breaked after the modification, which resulted in the better CAB of the MDFs. The FT-IR results indicated that the SDF had more hydroxyl groups, indicating that more cholesterol was combined and absorbed.

### Correlation analysis among the various properties of DFs

Pearson coefficients were calculated for the various properties of the DFs (Table 2). The pectin and polyphenol contents negatively correlated with the WHC, and the WSC and particle size positively correlated with the pectin content. The modification could destroy the cross-linked structures of polysaccharides from the cell wall and remove pectin, polyphenol and other components, which brought about an increased relative content of cellulose and smaller particle size. The SEM analysis indicated that the modification caused the breakage of the amorphous regions; thus, the crystallinities of the MDFs increased^30^. Therefore, the higher effective area of the fiber surface and the higher content of cellulose led to a higher WHC and WSC^14^. The WHC, OAC and WSC showed a positive correlation with each other, and the particle size showed a negative correlation with the WHC, OAC, and WSC, which indicated that a smaller particle size led to a higher WHC, WSC and OAC. The FT-IR results indicated that intramolecular hydrogen bonding interactions were broken and that the content of oligosaccharides was increased^30^. The MDFs showed weaker water evaporation peaks in the DSC curves and exhibited better microrheological properties than those of the DFs, with the properties correlated with the WSC and WHC. These results revealed that compared to unmodified DFs, the MDFs exhibited modified structures, crystallinity regions, and functional groups with better performance in terms of functional properties^13^. Therefore, these MDFs have potential applications in food and health products as functional ingredients.

**Table 2:** Pearson correlation coefficients among different properties of DFs from turnip.

	Yield	Pectin	Polyphenol	Protein	L*	a*	b*	$\Delta {\mathrm{E}}$	WHC	OAC	WSC	Crystallinity
Pectin	0.591*											
Polyphenol	0.310	0.732*										
Protein	− 0.576*	0.190	0.581*									
L*	− 0.601*	− 0.873*	− 0.310	0.151								
a*	0.354	0.812**	0.248	0.020	− 0.96**							
b*	0.457	0.538	− 0.178	− 0.439	− 0.880**	0.873**						
$\Delta {\mathrm{E}}$	1.000**	− 0.985**	− 0.999**	− 0.999**	− 1.000**	1.000**	1.000**					
WHC	− 0.999**	− 1.000**	− 0.999**	− 0.741	1.000**	− 1.000**	1.000**	0				
OAC	− 0.716**	− 0.102	0.427	0.949**	0.447	− 0.277	− 0.667*	− 1.000*	1.000**			
WSC	− 0.999**	− 1.000**	− 0.999**	− 0.741	1.000**	1.000**	1.000**	0	1.000**	1.000**		
Crystallinity	− 0.554	0.244	0.02	0.578*	− 0.329	0.579*	0.342	1.000**	1.000**	0.43	1.000**	
Particle size	0.609*	0.759**	0.116	− 0.320	− 0.979**	0.934**	0.954**	1.000**	− 0.998**	− 0.593*	− 0.998**	0.284

*P ≤ 0.05; **P ≤ 0.01.

## Conclusion

In conclusion, this study revealed changes in the compositional, structural, and functional properties of DFs. The SEM, FT-IR, XRD and DSC results showed that compared to the unmodified DFs, the MDFs had decreased particle sizes and more exposed functional groups that influenced their functional properties, such as •OH groups. The microrheological properties of the DFs showed that the MSDF network was structurally relaxed and that the MIDF network was structurally stable, which was verified by the structure analyses. Overall, our research suggests that turnip could be a good source of DFs and that MDFs have wider application prospects in food systems.

## Materials and methods

### Materials

White turnips were obtained from the Daliangshan region of Sichuan Province. The turnips were washed under running water and then sliced and immersed in 1.5% sodium chloride and 0.2% citric acid solution for 20 min. Next, the slices were put into a dry oven (GZX-9076MBE, Shanghai Boxun Industry & Commerce Co., Ltd., Shanghai, China) and dried at 60 °C. The dried slices were powdered by a pulverizer (SZ-500A-3, Yongkang Shanzhu Trading Co., Ltd., Zhejiang, China) and then sieved through an 80 mm sieve.

### Reagents

Mesophilic amylase (enzyme activity ≥ 100,000 U/g solid powder) was obtained from Jiangsu Ruiyang Biotechnology Co., Ltd., Suzhou, China. All reagents used in this work were of analytical reagent grade unless otherwise stated.

### Extraction of DFs

Turnip powders (20 g) were mixed with distilled water (1:4), and the pH was adjusted to 5.4 by using 5 mol/L NaOH. Then, mesophilic amylase (0.3%) was added to remove starch in a 65 °C water bath with stirring for 1 h. Next, the exact solution was mixed with 100 ml of 7% NaOH in a 65 °C water bath with continuous stirring for 45 min to remove protein. The mixture was centrifuged at 6000 rpm for 20 min (TG16G Changsha Yingtai Instrument Co., Ltd., Changsha, China). The obtained precipitate was first washed with distilled water and 95% ethanol, dried at 40 °C in a drying oven and then pulverized to obtain the IDF powder. For the supernatant, 4 volumes of 95% ethanol were added with gentle stirring, and then, the mixture was left overnight to precipitate SDF. The precipitate, which was obtained after filtering, was redissolved in distilled water (1:1) and then was lyophilized (SCIENTZ-10N Xinzhi Biotechnology Co., Ltd., Ningbo, China). The obtained powder was weighed to calculate the yield of SDF. The yields were calculated as follows:

$$ \begin{aligned} {\mathrm{The\,yield\,of\,SDF}}\,\left( \% \right) & = {\mathrm{the\,weight\,of\,dried\,SDF}}\,\left( {\mathrm{g}} \right)/{\mathrm{the\,weight\,of\,dried\,turnip\,powder}}\,\left( {\mathrm{g}} \right) \times 100\% \\ {\mathrm{The\,yield\,of\,IDF}}\,\left( \% \right) & = {\mathrm{the\,weight\,of\,dried\,IDF}}\,\left( {\mathrm{g}} \right)/{\mathrm{the\,weight\,of\,dried\,turnip\,powder}}\,\left( {\mathrm{g}} \right) \times 100\% \\ \end{aligned} $$

### Modification of IDF (Fig. 6)

IDF was modified to obtain the MIDF and MSDF^38^. IDF was soaked in 10% H_2_O_2_ (pH 11.5) with a solid–liquid ratio of 1:20 (g/mL) under ultrasound sonication at 200 W and 40 °C for 2 h (TH-400 BQ, Jining Tianhua Ultrasonic Electronic Instrument Co., Ltd., Shandong, China). Then, the mixture was centrifuged at 6000 rpm for 20 min. The obtained precipitate was then washed with distilled water and 95% ethanol and dried at 60 °C to obtain the MIDF. The supernatant was precipitated with 4 volumes of 95% ethanol and left overnight. The precipitate was then lyophilized to obtain the MSDF. The yields were calculated as follows:

$$ \begin{aligned} {\mathrm{The\,yield\,of\,MSDF }}\left( \% \right) & = {\mathrm{the\,weight\,of\,dried\,MSDF }}\left( {\mathrm{g}} \right)/{\mathrm{the\,weight\,of\,dried\,turnip\,powder}}\left( {\mathrm{g}} \right) \, \times 100\% \\ {\mathrm{The\,yield\,of\,MIDF }}\left( \% \right) \, & = {\mathrm{the\,weight\,of\,dried\,MIDF }}\left( {\mathrm{g}} \right)/{\mathrm{the\,weight\,of\,dried\,turnip\,powder }}\left( {\mathrm{g}} \right) \, \times 100\% \\ \end{aligned} $$

### Basic composition and granularity analysis

The moisture (method 925.09), protein (method 955.04) and ash (method 942.05) contents were measured by AOAC official methods^39^. The pectin content was measured by the carbazole colorimetric method. The polyphenol content was determined by the Folin–Ciocalteu method.

The average particle sizes of the DF samples were measured by a laser particle size analyzer (Nano ZS90, Malvern Instruments Ltd., England).

### Structure analyses

#### SEM

The microstructure of DFs was observed by a Zeiss EVO10 field emission scanning electron microscope (JSM-5610LV, JEOL, Japan). The dehydrated samples were placed on a metal stage and coated with a 10 nm gold layer.

#### FT-IR

DF was mixed with spectroscopic grade potassium bromide (KBr) powder and then pelletized to 1 mm. Four different DF samples were measured by a Fourier transform infrared spectrophotometer (FT/IR3000, Jusco, Japan) to obtain the FT-IR spectra from 400 to 4000 cm^−1^.

#### XRD

X-ray diffractometry was performed on a Bruker D8 Advance X-ray diffractometer (Bruker, AXS, Germany) with Cu radiation at 40 kV and an incident current of 30 mA. The angular region ranging from 5° to 70° was scanned with a step length of 0.02° and a step rate of 0.2 s/step. The degree of crystallinity was calculated using the MDI Jade 5.0 software (Materials Data, Inc., California, USA)^13^.

#### DSC

The thermal properties of DFs were analyzed using a differential scanning calorimeter (DSC1, Mettler Toledo, USA). A sample (5–10 mg) was placed into an aluminum pan and immediately covered with a aluminum cover. The calorimeter was calibrated using indium with an empty aluminum pan used as a reference. The sample pans were heated from 25 °C to 400 °C at a rate of 10 °C/min^30^.

### Physical properties

#### Color measurement

The color characteristics (L*, a* and b*) of DFs were determined using a colorimeter (NR20XE, 3nh Co., China), and the results were expressed in terms of the CIELAB system.

#### WHC

The WHC of DF was measured by the method of Sowbhagya et al. (2007)^40^. DF (IDF and MIDF) powder (0.04 g) was hydrated in 3 mL of distilled water at 25 °C for 2 h and then centrifuged at 3000 rpm for 20 min. Then, the fresh weight of the residue was recorded. The WHC was calculated by the following equation:

$$ {\mathrm{WHC}}\left( {\mathrm{g/g}} \right) \, = \, \left( {{\mathrm{m}}_{{\mathrm{f}}} - {\mathrm{ m}}} \right)/{\mathrm{m}} $$

where m_f_ is the weight of the fresh residue (g) and m is the dry weight of the sample powder (g).

#### WSC

The WSC was calculated by the method of Sowbhagya et al. (2007) with some modifications^40^. DF (IDF and MIDF) powder (0.2 g) was hydrated in 10 mL of distilled water in a graduated test tube at 25 °C for 24 h. The volume of DFs was recorded, and the WSC was calculated by the following equation:

$$ {\mathrm{WSC}}\left( {\mathrm{mL/g}} \right) = \left( {{\mathrm{v}}_{1} - {\mathrm{v}}_{0} } \right)/{\mathrm{w}}_{0} $$

where v_1_ is the volume of the hydrated DF, v_0_ is the volume of the DF before hydration, and w_0_ is the weight of the DF before hydration.

#### OAC

The OAC was calculated by the method of Abdul-Hamid and Luan (2000) with some modifications^41^. DF (IDF, SDF, MIDF and MSDF) powder (0.12 g) was mixed with 3 mL of soya bean oil for 1 h at 37 °C. The mixture was centrifuged at 3000 rpm for 20 min. The OAC was determined based on the amount of soya bean oil retained by the DF:

$$ {\mathrm{OAC}}\left( {\mathrm{g/g}} \right) = \left( {{\mathrm{m}}_{{\mathrm{r}}} {-}{\mathrm{m}}} \right) \times 100/{\mathrm{m}} $$

where m_r_ is the residue weight, which contained the oil (g), and m is the original weight of the DF (g).

#### Cation exchange capability

The cation exchange capability was calculated by the method of Chau and Huang (2003) with slight modifications^33^. DF (IDF, SDF, MIDF and MSDF) powder (0.3 g) was dissolved in 30 mL of 0.01 mol/L HCl and was refrigerated at 4 °C overnight, which was set as the control group. After the temperature reached 25 °C, 0.5 mL of 0.1 mol/L NaOH was added to the mixture to achieve complete cation exchange, and the pH change of the solution was recorded.

#### Microrheological behavior

A commercial Rheolaser Master (Formulation, I'Union, France), which was based on diffusing-wave spectroscopy (DWS), was used for measuring the microrheology of the DFs. This technique is similar to dynamic light scattering and measures the Brownian motion of particles, which depends on the viscoelastic structure of the sample. Aqueous solutions of 40% (w/v) SDF and MSDF and 5% (w/v) suspensions of IDF and MIDF were measured at 25 °C. The instrument measures Brownian motion of the particle as the droplet mean square displacement (MSD) versus time. MVI parameters of the samples were obtained with the software RheoSoft Master 1.4.0.0^35^.

#### CAB

The cholesterol absorption abilities of the samples were determined according to a previous study^3^. First, a 0.5 g sample (IDF or MIDF) or 0.1 g sample (SDF or MSDF) was dissolved in 25 mL of 1 mg/mL cholesterol solution. The reaction mixture was incubated in a water bath at 37 °C for 2 h, followed by centrifugation (4000 × g for 20 min). Then, the cholesterol content of 0.1 mL of supernatant was tested by the phthalic aldehyde method at 550 nm.

### Statistical analyses

Each experiment was carried out in triplicate, and the data are expressed as the mean ± standard deviation (SD). Pearson correlation coefficients (r) were calculated for the various physicochemical properties, and the least significant difference (LSD) test was compared by SPSS 10.0 software; *P* < 0.05 was considered statistically significant.
